# Cortical Mechanisms Underlying Immersive Interactive Virtual Walking Treatment for Amelioration of Neuropathic Pain after Spinal Cord Injury: Findings from a Preliminary Investigation of Thalamic Inhibitory Function

**DOI:** 10.3390/jcm12175743

**Published:** 2023-09-04

**Authors:** Sylvia M. Gustin, Mark Bolding, William Willoughby, Monima Anam, Corey Shum, Deanna Rumble, Victor W. Mark, Lucie Mitchell, Rachel E. Cowan, Elizabeth Richardson, Scott Richards, Zina Trost

**Affiliations:** 1 NeuroRecovery Research Hub, School of Psychology, University of New South Wales, Sydney 2052, Australia; 2 Centre for Pain IMPACT, Neuroscience Research Australia, Sydney 2031, Australia; 3 Department of Radiology, University of Alabama at Birmingham, Birmingham, AL 35233, USA; 4 Department of Psychiatry and Behavioral Neurobiology, University of Alabama at Birmingham, Birmingham, AL 35233, USAnoriscat.mitchell@gmail.com (L.M.); 5 Immersive Experience Laboratories LLC, Birmingham, AL 35203, USA; 6 Department of Psychology and Counseling, University of Central Arkansas, Conway, AR 72035, USA; 7 Department of Physical Medicine & Rehabilitation, University of Alabama at Birmingham, Birmingham, AL 35233, USA; 8 Department of Behavioral & Social Sciences, University of Montevallo, Montevallo, AL 35115, USA; 9 Department of Psychological and Brain Sciences, Texas A&M University, College Station, TX 77843, USA

**Keywords:** virtual reality, spinal cord injury neuropathic pain, γ-aminobutyric acid, thalamus, MR spectroscopy

## Abstract

Background: Neuropathic pain following spinal cord injury (SCI) affects approximately 60% of individuals with SCI. Effective pharmacological and non-pharmacological treatments remain elusive. We recently demonstrated that our immersive virtual reality walking intervention (VRWalk) may be effective for SCI NP. Additionally, we found that SCI NP may result from a decrease in thalamic γ-aminobutyric-acid (GABA), which disturbs central sensorimotor processing. Objective: While we identified GABAergic changes associated with SCI NP, a critical outstanding question is whether a decrease in SCI NP generated by our VRWalk intervention causes GABA content to rise. Method: A subset of participants (*n* = 7) of our VRWalk trial underwent magnetic resonance spectroscopy pre- and post-VRWalk intervention to determine if the decrease in SCI NP is associated with an increase in thalamic GABA. Results: The findings revealed a significant increase in thalamic GABA content from pre- to post-VRWalk treatment. Conclusion: While the current findings are preliminary and should be interpreted with caution, pre- to post-VRWalk reductions in SCI NP may be mediated by pre- to post-treatment increases in thalamic GABA by targeting and normalizing maladaptive sensorimotor cortex reorganization. Understanding the underlying mechanisms of pain recovery can serve to validate the efficacy of home-based VR walking treatment as a means of managing pain following SCI. Neuromodulatory interventions aimed at increasing thalamic inhibitory function may provide more effective pain relief than currently available treatments.

## 1. Introduction 

Approximately 60% of individuals develop neuropathic pain following spinal cord injury (SCI NP). This type of pain is experienced at or below the zone of injury and is described as sharp, burning, and unbearable [1]. It remains persistent and intensifies progressively over time [2,3]. Although pharmacological agents (e.g., serotonin- and noradrenaline-reuptake inhibitors, antiepileptics, and tricyclic antidepressants) are treatment mainstays [4], none of them have consistently proven to be comprehensively effective [5,6,7,8]. Furthermore, many pain medications are associated with numerous negative side effects, such as somnolence, sleep disturbances, blurred vision, addiction, abuse, and toxicity [9,10,11]. Non-pharmacological therapies, such as repetitive transcranial magnetic stimulation, cranial electrotherapy stimulation, transcutaneous electrical nerve stimulation, and psychological interventions, have minimal negative side effects but result in only nominal reductions in pain [12]. As a result, many people with SCI experience ongoing neuropathic pain with no access to effective treatment.

Recently, we demonstrated that an immersive, interactive virtual walking intervention may be an effective treatment for unremitting SCI NP [13]. We pioneered an immersive, interactive virtual reality walking intervention (VRWalk) as a novel extension to visual feedback/illusory walking therapies that have previously been shown to reduce SCI NP [1,14,15,16,17]. Through our VRWalk interface, individuals with SCI can, for the first time, freely control their own virtual gait and interact by their own volition with a fully immersive virtual environment. In our study, we compared VRWalk to a passive, non-interactive walking treatment (analogous to previous feedback/illusory walking therapies) in people with SCI NP [13]. Participants in the interactive condition (VRWalk) showed a significant decrease in SCI NP intensity and interference pre- to post-intervention compared with the passive condition [13]. 

The significant analgesic improvement in participants who experienced VRWalk may be explained by changes occurring in contributing cortical mechanisms. For example, perceiving an environment in an immersive, first-person view activates sensorimotor brain regions more adaptively compared with passive, third-person perspectives [18,19]. This finding is important since SCI NP is associated with structural, functional, and biochemical changes in cortical areas important for sensorimotor processing, such as the thalamus, primary somatosensory cortex (S1), and motor cortex (M1) [20,21,22,23]. Animal models have demonstrated the role of thalamic γ-aminobutyric acid (GABA)-ergic processes in inhibiting pain [24,25,26]. Although data from human studies exhibited a cross-over design, it suggests that a decrease in GABA levels within the thalamic reticular nucleus (TRN) may disturb central sensorimotor processing, which in turn may result in SCI NP [22]. While we have identified thalamic neurochemical changes associated with SCI NP, a critical outstanding question is whether the decrease in SCI NP generated by our novel immersive, interactive VR treatment (VRWalk) is associated with a subsequent rise in GABA levels. To address this question, a subset of participants enrolled in the VRWalk trial [13] underwent magnetic resonance (MR) spectroscopy pre- and post-intervention to determine if the decrease in SCI NP is associated with an increase in thalamic GABA content. 

## 2. Materials and Methods

### 2.1. Study Design

Single-arm study of neuroimaging data.

### 2.2. Participants

A pilot subset of seven participants from the interactive condition of the VRWalk trial [13] were randomly selected from the larger cohort and underwent GABA spectroscopy before and after the Interactive Virtual Walking condition at the University of Alabama at Birmingham (UAB) Highlands Hospital. We followed a simple random selection. The research assistant chose a name from the list of interactive VRWalk participants. Pilot funding from the UAB Department of Radiology facilitated imaging of the seven participants. Recruitment procedures, as well as inclusion and exclusion criteria, are fully outlined elsewhere [13]. To summarize, participants from the UAB SCI Model System of Care were included if they had complete paraplegia, as classified by the International Standards for Neurological Classification of Spinal Cord Injury (ISNCSCI). Participants were also included if they endorsed two or more items on the 4-item Spinal Cord Injury Pain Instrument (SCIPI), which has a strong overlap with clinical diagnoses of SCI NP [27]. Items 1–3 of the SCIPI pertain to pain descriptors commonly associated with SCI NP, while Item 4 identifies pain experienced in insensate areas. Eligible participants were between the ages of 18–65, had a diagnosed SCI for at least one year, experienced persistent SCI NP (for 3 months or longer) with a minimum severity of 4/10 on a Numeric Rating Scale (NRS), and had no changes in their pain medication regimen in the past month. Participants were excluded from participating in the VRWalk trial if they had a history of moderate to severe brain injury or severe psychiatric disorder [13] and were further excluded from the present study if they had any contraindication to MRI (e.g., implanted metal clips). 

### 2.3. Regulatory Approvals

The VRWalk study protocol and imaging procedures were approved by the UAB Institutional Review Board (IRB-300001463, 3 May 2019), and all participants provided informed written consent. The VRWalk trial was registered on ClinicalTrials.gov (Identifier: NCT03735017; date of first registration: 8 November 2018). The VRWalk trial was a non-randomized single-arm trial; thus, blinding was not implemented.

### 2.4. Procedures

Prior to imaging and engaging in the VRWalk intervention, baseline pain intensity interference and disability measures were collected. Participants traveled to UAB to undergo MR spectroscopy prior to starting the VRWalk interactive condition. Following baseline scanning, participants returned to their residences, where they participated in the intervention. Research assistants traveled daily to participants’ homes to set up the VR equipment for each session. Participants engaged in two separate 10 min VR gameplay sessions per day, with a minimum of 4 h between sessions, resulting in a total of 20 min of daily VR intervention over a span of 10 days within a two week period. Procedural details regarding delivery schedules have been described previously [13]. Pain intensity, interference, and disability measures were collected again after the last VRWalk session, and participants were brought back to UAB for post-intervention MR spectroscopy scanning. 

### 2.5. Immersive Interactive Virtual Walking Interface

**Figure 1:**
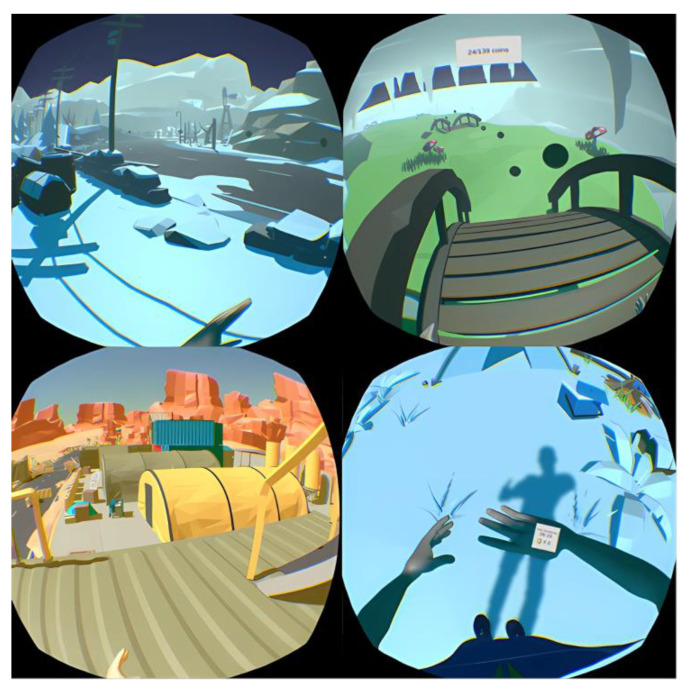
In-game graphics from three open virtual worlds.

The VR game used in the VRWalk trial, developed by Immersive Experience Labs (IXL), used a cross-platform game engine (Unity Game Engine) and was made available for Windows PC devices. The VR game was hosted on digital distribution software, allowing participants’ progression through the VR environment and gameplay to be saved between VR sessions over the entire intervention period. The immersive VR environment consisted of a first-person view from an avatar that could be customized to match participants’ own physical characteristics (e.g., skin tone, weight, etc.). A research assistant oversaw each VRWalk session at participants’ individual residences, including the research assistant configuring the VR equipment before each session, which is further described elsewhere [13]. The avatar and VR environment were presented to participants via an HTC Vive^®^ Head Mounted Display (HMD) connected to a laptop computer. HTC Vive^®^ includes handheld controllers with accelerometers that capture actual movement to convert to virtual movement in the 3D VR environment (Figure 1). The HTC Vive^®^ has native a frames per second (fps) rate of 90 fps. The rendering of the environment averaged ~60 fps, which was interpolated with the participant’s motion to present 90 fps. In the interactive condition of the VRWalk trial, hand controllers tracked arm movements that were translated into movement of the virtual lower extremities. Progression through the virtual world was incentivized using limited monetary rewards. Details regarding gameplay incentives and how they were awarded have been described previously [13]. 

### 2.6. Measures

#### 2.6.1. Chronic Pain Measures

##### Pain Intensity

Participants’ pain intensity was measured via a 0–10 Numeric Rating Scale (NRS), with anchors of 0 = “no pain” and 10 = “worst possible pain” [28,29]. The NRS is a psychometrically sound and frequently used measure that reliably captures changes in pain across time [30,31,32,33] and is a recommended measure to use in clinical trials assessing pain as an outcome [34]. In the present study, participants were asked to provide an average NRS rating over the past week before the VRWalk intervention and an average NRS rating one week following the completion of the 10 day intervention. Participants also completed a visual analog scale (VAS [35], 0 = “no pain” and 100 = “worst possible pain”) to rate their current level of pain immediately prior to and following the 10 days of intervention. Both NRS and VAS were administered and completed by paper and asked specifically about participants’ below-level neuropathic pain. 

##### Pain Disability Index 

The Pain Disability Index (PDI) [36] measures the degree of self-reported pain-related disability. Seven items are assessed on a 0–10 NRS in which 0 means no disability, and 10 is maximum disability. The sum of the seven items equals the total score of the PDI, which ranges from 0 to 70, with higher scores reflecting more pain-related disability. The PDI was assessed prior to and following the VRWalk intervention. 

##### Pain Interference

Pain interference describes the extent to which pain restricts or disrupts individuals’ physical, mental, and social activities [37]. Pain Interference was assessed using an NRS to measure how much neuropathic pain interfered with day-to-day activities in the last week, ranging from 0 (No Interference) to 10 (Extreme Interference). Pain interference was assessed prior to and after the VRWalk intervention.

##### Neuropathic Pain Scale 

The Neuropathic Pain Scale (NPS) [38] was used to measure the severity of SCI NP symptoms. The NPS encompasses eight items addressing specific NP qualities rated on a 0 to 10 scale (e.g., “not burning” to “the most burning sensation imaginable”). The NPS has good psychometric properties and is recommended for measuring change in SCI-NP in clinical trials [33]. A composite sum of the eight items was used, with higher scores indicating greater severity of SCI-NP. The NPS was collected before and after the VRWalk intervention.

### 2.7. MR Spectroscopy Measures

#### GABA-Edited MEscher–GArwood Point RESolved Spectroscopy (MEGA-PRESS) Spectra

**Figure 2:**
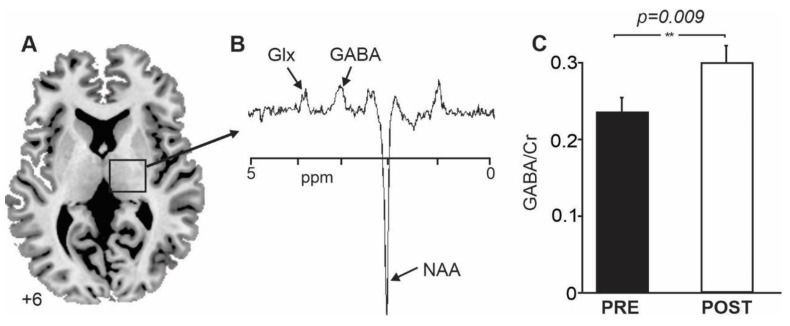
(**A**) Axial slice showing the location from which GABA-edited MEGA-PRESS spectroscopy was performed in the right thalamus of participants. The slice location in the Montreal Neurological Institute space is indicated at the lower left of the image. (**B**) Typical MEGA-PRESS spectrum obtained from the thalamus. (**C**) A plot of the mean (±SD) GABA/creatine ratios in the thalamus of participants prior to and following 10 days of Immersive Interactive Virtual Walking Treatment. Glx: glutamine; GABA: gamma-aminobutyric acid; Cr: Creatine; ppm: parts per million.

Participants laid supine headfirst on the bed of a 3T MRI system (Siemens MAGNETOM Prisma) with their heads immobilized in a 32-channel head coil. A T1-weighted, magnetization-prepared rapid gradient echo (MPRAGE) 3D imaging sequence with 1 mm isotropic voxels was acquired. We used multi-planar reformats (axial, sagittal, coronal) for voxel placement. GABA-edited MEGA-PRESS spectra were acquired from a voxel (20 × 20 × 20 mm^3^) centered in each participant in the right thalamus (Figure 2). While the voxel covered the entire thalamus, the TRN contains nearly all of the GABAergic neurons within the thalamus [39]; thus, any measured GABA content is located in the TRN. The GABA-edited MEGA-PRESS sequence parameters were as follows: repetition time (TR) = 2000 ms, echo time (TE) = 68 ms, 256 averages, and total acquisition time: 20 min. One hundred twenty eight averages were acquired with the MEGA pulse centered at 1.9 ppm (ON) and 128 averages with the pulse centered at 7.5 ppm (OFF). We performed manual shimming, which resulted in line widths of <10 Hz for all spectra. The Siemens Brain Dot Engine auto-align function was used to ensure consistency in the follow-up voxel placement of the same participant and from participant to participant.

### 2.8. Analysis

#### 2.8.1. GABA-Edited MEGA-PRESS Spectral Analysis

The acquired spectra were analyzed using the Java-based magnetic resonance user interface (jMRUI 6.0, European Union project). We summed the ‘‘ON’’ and ‘‘OFF’’ spectral subsets to produce single ‘‘ON’’ and ‘‘OFF’’ 68 ms sub-spectra for each spectra dataset. These 68 ms sub-spectra were then subtracted, resulting in GABA-edited MEGA-Press difference spectra to measure GABA concentration at 3.01 ppm. The GABA-edited MEGA-PRESS difference spectra were phased with respect to both the zero- and first-order phases. GABA was quantified using AMARES, a nonlinear least-square fitting algorithm operating in the time domain. Peak fitting for GABA was performed after manually defining the center frequency and line width of the GABA peak and modeling the GABA peak as a singlet. We used Lorentzian curves to obtain the peak amplitude for this resonance.

The ‘‘OFF’’ spectral subsets were summed, producing a single ‘‘OFF’’ 68 ms sub-spectra for each spectra dataset to measure creatine concentration at 3.02 ppm. The single ‘‘OFF’’ 68 ms sub-spectra was t-phased with respect to both the zero- and first-order phases. Spectral fitting in Advanced Method for Accurate, Robust, and Efficient Spectral Fitting (AMARES) was performed after manually defining the center frequency and line width of the creatine peak and modeling the creatine peak as a singlet. AMARES represents an enhanced technique to precisely and efficiently estimate the parameters of noisy magnetic resonance spectroscopy (MRS) signals in the time domain. We calculated ratios for GABA relative to creatine. 

#### 2.8.2. Spectral Quality Assessment 

We calculated the variances from the peak areas and the standard deviations of the fit for GABA in each participant to assess the goodness of fit. Average line widths and signal-to-noise ratios (SNRs) were also examined. We assessed SNRs using the peak amplitudes of N-acetylaspartate (NAA) in the GABA OFF spectrum compared with the peak amplitude of the noise from a signal-free section of the spectrum of approximately 10 ppm in each participant. 

#### 2.8.3. Data Analysis

We calculated means and standard deviations for NRS, VAS, and GABA/creatine ratios. Paired sample t-tests were used to examine alterations in pain intensity and GABA/creatine ratios measured before and after the VRWalk intervention. Significant correlations between pain and neurochemical data were examined through Pearson correlations. SPSS version 26.0 (IBM SPSS Statistics) was used to perform the statistical analyses. A significance level of *p* < 0.05 was used across this study.

## 3. Results

### 3.1. Pain Intensity

**Figure 3:**
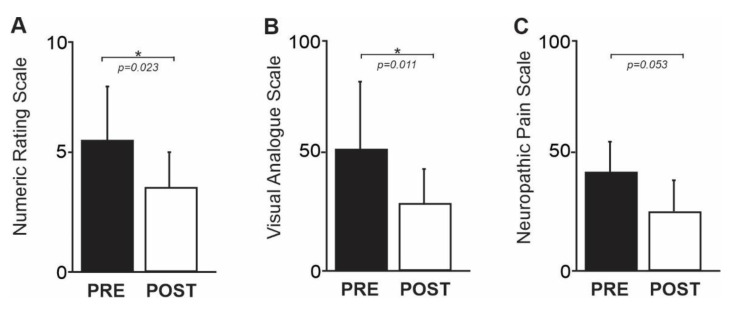
(**A**) A plot of the mean (±SD) numeric rating scale (NRS) rating over the preceding week before the VRWalk intervention as well as approximately one week following the completion of the 10 day intervention. (**B**) A plot of the mean (±SD) visual analog scale (VAS) values of current pain intensity of participants prior to and after 10 days of VRWalk intervention. (**C**) A plot of the mean (±SD) neuropathic pain scale (NPS) ratings of pain severity prior to and after 10 days of VRWalk intervention.

The demographics and pain characteristics of the sample are shown in Table 1. Although the time since injury varied, all participants had a complete thoracic spinal cord injury. Participants showed a significant decrease in NRS ratings of average pain collected prior to and following the intervention (mean NRS ± SD PRE Intervention: 5.6 ± 2.4; mean NRS ± SD POST Intervention: 3.4 ± 1.7; t = −0.037, df = 6, *p* = 0.023; Figure 3, individual NRS values are represented in Table 2). Participants also showed a significant decrease in VAS ratings of pain collected prior to and after the intervention (mean VAS ± SD PRE Intervention: 52 ± 31; mean VAS ± SD POST Intervention: 22 ± 19; t = 3.648, df = 6, *p* = 0.011; Figure 3, individual VAS values are represented in Table 2). There was a decrease in NPS ratings of pain severity from pre- to post-intervention that approached significance (mean PDI PRE Intervention: 19 ± 10; mean PDI POST Intervention ± SD POST Intervention: 21 ± 20; t = −0.486, df = 6, *p* = 0.644; mean Pain Interference PRE Intervention: 2.1 ± 2.3; mean Pain Interference POST Intervention ± SD POST Intervention: 0.6 ± 0.8; t = 3.161, df = 6, *p* = 0.052). Participants showed a marginal trend toward a significant decrease in NPS ratings of pain severity collected prior to and following the intervention (mean NPS ± SD PRE Intervention: 40.6 ± 15; mean NRS ± SD POST Intervention: 25.0 ± 16; t = 2.408, df = 6, *p* = 0.053; Figure 3, individual NPS values are represented in Table 2). None of the seven participants reported any adverse effects related to the VRWalk intervention.

### 3.2. GABA-Edited MEGA-PRESS Spectroscopy

Participants showed a significant decrease in mean GABA/creatine ratios from pre- to post-VRWalk intervention (mean GABA/creatine ratio ± SD PRE Intervention: 0.24 ± 0.022; mean GABA/creatine ratio ± SD POST Intervention: 0.3 ± 0.034; t = −3.825, df = 6, *p* = 0.009; Figure 1, individual GABA/creatine ratios are represented in Table 2). However, there was no significant linear relationship between the change in GABA/creatine ratios and the change in pain intensity from pre- to post-intervention (NRS: r = −0.282, *p* = 0.3; VAS: r = −0.417, *p* = 0.2, Table 3). There was also no significant linear correlation between pre-intervention GABA/creatine ratios and pre-intervention NRS ratings (r = −0.2, *p* = 0.3, Table 3) and pre-intervention GABA/creatine ratios and participants’ pain duration (r = −0.4, *p* = 0.2, Table 3). Though the Pearson correlation coefficient was of moderate strength between pre-intervention GABA/creatine ratios and pre-intervention VAS ratings, it did not reach significance (r = 0.5, *p* = 0.1, Table 3). Similarly, there was a positive linear correlation between post-intervention GABA/creatine ratios and post-intervention NRS ratings; this relationship did not reach the statistical threshold for significance (r = 0.6, *p* = 0.1, Table 3). Lastly, there was no significant relationship between post-intervention GABA/creatine ratios and post-intervention VAS ratings (r = −0.2, *p* = 0.3, Table 3).

Two of the seven participants were treated with baclofen and gabapentin (Table 1). Both participants’ medication regimens were constant across the intervention. When both participants were excluded from the paired t-test analysis, the results still showed a significant decrease in mean GABA/creatine ratios from pre- to post-VRWalk intervention (mean GABA/creatine ratio ± SD PRE Intervention: 0.23 ± 0.016; mean GABA/creatine ratio ± SD POST Intervention: 0.3 ± 0.032; t = −7.364, df = 4, *p* = 0.002). 

### 3.3. Spectral Quality Assessment

Line widths, SNRs, and variances of GABA and creatine were all well within acceptable limits for data quality according to the consensus on clinical MRS of the brain [40,41]. Line widths for all spectra were <10 Hz after manual shimming. Furthermore, there was no significant difference in the mean-variance of GABA (%) between pre- and post-intervention (GABA variance mean ± SD: PRE Intervention: 17.4 ± 4.5% (minimum 13.12; maximum 26.17); POST Intervention: 18.1 ± 4.1% (minimum 13.0; maximum 23.06); t = −0.305, df = 6, *p* = 0.77). There was also no significant difference in the mean-variance (%) of creatine between pre- and post-intervention (creatine variance mean ± SD: PRE Intervention: 5.0 ± 0.67% (minimum 4.18; maximum 5.26); POST Intervention: 5.00 ± 0.61% (minimum 4.40; maximum 5.95); t = −0.037, df = 6, *p* = 0.97). Finally, there was no significant difference in mean SNR ratios between before and after intervention (SNR ratios mean GABA spectra: PRE Intervention ± SD: 11.0 ± 2.2 (minimum 8.83; maximum 13.25); POST Intervention: 11.4 ± 3.7 (minimum 3.28; maximum 13.3); t = −0.339, df = 6, *p* = 0.75).

## 4. Discussion 

This study demonstrated that individuals with SCI NP have a significant change in thalamic GABA content pre- compared with post-VWalk treatment. That is, individuals with SCI NP have significantly increased GABA/creatine ratios following a 10 day immersive, interactive virtual walking therapy compared with pre-intervention. Participants also reported a significant decline in SCI NP intensity following VRWalk treatment [13], though there was no significant correlation between GABA/creatine ratios and SCI NP intensity as well as pain duration, as evidenced by the present pilot results. Nevertheless, these results indicate an association between SCI NP and neurotransmitter dysregulation in the thalamus, consistent with similar dysregulation observed in other central nervous system regions, such as the medial prefrontal cortex in SCI NP [42,43]. 

While speculative in the context of the current finding, this plausibly aligns with a cortical model of disinhibition in which the cortical inability to suppress pain potentially underlies the experience of SCI NP [44]. In line with this model, we suggested that a decrease in the inhibitory neurotransmitter GABA in the thalamic reticular nucleus (TRN) results in an altered thalamocortical connection between the TRN and the sensorimotor cortex [22]. The disruption of TRN—sensorimotor cortex connection may lead to functional changes within the sensorimotor cortex. For instance, individuals experiencing SCI NP have shown reorganization in the primary somatosensory (S1) cortex, leading to a diminution of activity in regions related to innervating the legs and a rearrangement of cortical representation for other body regions (i.e., the little finger and thumb) toward regions normally associated with innervating the legs [23]. Additionally, individuals with SCI NP exhibit functional alterations in the primary motor cortex (M1) when engaging with imagery of lower extremity movement, resulting in modulation of SCI NP through neural processes or neuromodulation [21]. Notably, the extent of these sensorimotor functional changes significantly relates to SCI NP intensity [21,23], making these functional alterations a potential target for intervention. Indeed, evidence suggests visual feedback interventions may activate sensorimotor areas in the brain, resulting in decreased SCI NP [1,14,15,16,17,30]. Thus, visual illusion modalities, such as virtual walking, may activate the sensorimotor cortex in an adaptive manner with the consequence that SCI NP is reduced. Moreover, incorporating a first-person perspective to such modalities, as was performed in the VRWalk intervention, may engage cortical motor networks to a greater extent than third-person perspectives [18,19]. These effects could potentially be amplified by the heightened presence and immersion achieved from a first-person perspective [45], which are characteristics of VR paradigms previously associated with increased reductions in pain [46,47,48]. 

Although preliminary, the results of the current study indicate that creating a completely immersive experience of walking in a normal gait, as achieved in the VRWalk intervention, could potentially activate cortical regions associated with sensorimotor execution and control (i.e., S1 and M1). This, in turn, might positively influence the corticothalamic circuit, resulting in the stabilization of neurotransmitter dysregulation associated with pain, as evident in the elevated thalamic GABA content. Although we did not measure S1 and M1 activation in this study, participants reported a sensation of performing and sensing a walking motion [13], providing anecdotal evidence that both S1 and M1 might have been activated in an adaptive manner. In line with this argument, evidence shows that both real and illusory sensorimotor stimulation results in a decrease in neuropathic pain by targeting the sensorimotor cortex, which has been previously found to functionally reorganize following SCI and amputation [14,16,49]. 

The specific cortical mechanisms underlying pain relief of illusory sensorimotor stimulation remain unclear. For example, any temporal causal relationship that exists between illusory sensorimotor cortex stimulation via the immersive VRWalk intervention and sensorimotor cortex activation, thalamocortical connection, and thalamic GABA concentration is not evident from this study. Nonetheless, this preliminary study indicates that further investigation of changes in thalamic GABA concentrations and its role in normalizing inhibitory function is potentially a fruitful line of future research. Specifically, future studies with larger samples are needed to determine the potential mediating role of changes in thalamic GABA on SCI NP reductions following the VRWalk intervention, as well as its role in reversing maladaptive sensorimotor cortex reorganization associated with chronic pain.

## 5. Limitations

The current study was pilot in nature, and while it provides direction for future research, it lacks a sufficient sample size to infer true population effects from these results alone. The small sample size also prevented the control of individual differences in medication types (i.e., two out of seven participants were treated with baclofen and gabapentin), even though participants’ medication regimens remained constant during the intervention. Despite this, the results still demonstrated a significant decrease in mean GABA/creatine ratios from pre- to post-VWalk intervention, even after excluding the two participants on pain medications. Nevertheless, it is important to consider that certain SCI NP medications, such as anticonvulsants, may impact GABAergic systems [50]. Future research investigating neurotransmitter mechanisms underlying pain reduction should take this into account. Furthermore, since all seven participants were exposed to the interactive VRWalk intervention, there is no comparator group to definitively rule out any effect of time on changes in thalamic GABA concentrations and pain intensity or to account for other potential confounding factors. Thus, we cannot fully determine whether the observed reduction in GABA content and pain intensity was due to the intervention or other factors. This study also lacks an able-bodied control or non-SCI chronic pain comparator group, which would clarify whether changes in GABA/creatine ratios following VRWalk intervention are unique to those with SCI-NP. Due to the lack of follow-up scanning sessions, we were not able to determine if both the decrease in pain intensity and the increase in GABA content persisted over time. Future studies should incorporate follow-up measurements to investigate whether the effects of 10 days of interactive VRWalk treatment on pain intensity and GABA content extend beyond the intervention period. 

## 6. Conclusions

The current study advances existing research on illusory walking treatment for SCI NP by providing first hints about the action of immersive virtual reality treatment on the human brain. While caution should be used when interpreting these preliminary results, they nonetheless suggest that immersive virtual walking may alleviate SCI NP through the normalization of thalamic neurotransmitter dysregulation. Future studies are called for to better understand and confirm causal mechanisms. GABAergic changes may play a role in a larger model of cortical change associated with virtual reality walking that involves activating the sensorimotor cortex. Neuromodulative interventions aimed at increasing thalamic inhibitory function may provide more effective pain relief than currently available treatments.

## Figures and Tables

**Table 1:** Participant Demographic and Pain Characteristics.

Code	Sex	Age	ASIA ISNCSCI Grade	Level of Injury	Years Since Injury	Pain Duration (Years)	Pain Location	Pain Level	Pain Medication
1	M	23	A	T7	5	5	Bilateral back, feet	Below	None
2	M	35	A	T7	15	15	Bilateral feet, shins	Below	None
3	M	36	A	T12	10	10	Left buttocks, left lower back	Below	None
4	M	48	A	T12	6	6	Bilateral toes	Below	Baclofen, gabapentin
5	M	48	A	T1	14	14	Bilateral buttocks, feet	Below	None
6	M	56	A	T10	11	11	Bilateral toes, upper legs	Below	Baclofen, gabapentin
7	M	70	A	T11–12	4	4	Bilateral abdomen, legs	Below	None

Note. M = male; ASIA ISNCSCI = International Standards for Neurological Classification of Spinal Cord Injury American Spinal Injury Association; Neurological level: T = thoracic level.

**Table 2:** Individual GABA/creatine ratios, numeric rating scale (NRS), and visual analog Scale (VAS) values.

Code	Pain Duration (Years)	GABA/Cr (ppm) PRE Therapy	GABA/Cr (ppm) POST Therapy	NRS PRE Therapy	NRS POST Therapy	VAS PRE Therapy	VAS POST Therapy	NPS PRE Therapy	NPS POST Therapy
1	5	0.25	0.31	6	2	30	8	24	11
2	15	0.21	0.27	9	6	85	48	63	15
3	10	0.24	0.36	8	5	80	13	55	56
4	6	0.28	0.26	3	3	77	50	27	10
5	14	0.21	0.27	6	3	55	15	46	21
6	11	0.24	0.29	4	1	3	1	36	28
7	4	0.22	0.31	3	4	32	21	33	34
Mean (±SD)	9.3 ± 4.4	0.24 ± 0.022	0.30 ± 0.034	5.6 ± 2.4	3.4 ± 1.7	52 ± 31	22 ± 19	40.6 ± 15	25 ± 16

Note. Cr = Creatine; NRS = Numeric Rating Scale; VAS = Visual Analogue Scale; ppm = ppm: parts per million; GABA = gamma-aminobutyric acid; NPS = Neuropathic Pain Scale.

**Table 3:** Correlations.

Variable 1	Variable 2	Correlation Coefficient (r)	Significance Level (*p*)
Change in GABA/creatine ratios from pre- to post-intervention	Change in pain intensity (NRS) from pre- to post-intervention	−0.282	0.3
Change in GABA/creatine ratios from pre- to post-intervention	Change in pain intensity (VAS) from pre- to post-intervention	0.417	0.2
Pre-intervention GABA/creatine ratios	Pre-intervention NRS ratings	−0.2	0.3
Pre-intervention GABA/creatine ratios	Pain duration	−0.04	0.2
Pre-intervention GABA/creatine ratios	Pre-intervention VAS ratings	0.5	0.1
Post-intervention GABA/creatine ratios	Post-intervention NRS ratings	0.6	0.1
Post-intervention GABA/creatine ratios	Post-intervention NRS ratings	−0.2	0.3

Note. Cr = NRS = Numeric Rating Scale; VAS = Visual Analogue Scale; GABA = gamma-aminobutyric acid.

## Data Availability

The datasets generated and/or analyzed during the current study are available from the corresponding author upon reasonable request.
